# Metabolic profiling of severe early childhood caries and black stain: pathway and biomarker discovery in 3-6-year-olds

**DOI:** 10.3389/fmolb.2026.1838384

**Published:** 2026-08-21

**Authors:** Ying Chen, Jing Xie, Li Chen, Xuezhen Peng, Guicong Ding, Guo-bing Wang, Li Zhang

**Affiliations:** 1 Department of Stomatology, Shenzhen Children’s Hospital, Shenzhen, Guangdong, China; 2 School of Dentistry, Shenzhen University Medical School, Shenzhen, China; 3 Shenzhen Pediatrics Research Institute, Shenzhen Children’s Hospital, Shenzhen, China

**Keywords:** dental caries, machine learning predictions, network interaction, supragingival plaque, untargeted metabolomics, weighted gene co-expression network

## Abstract

The present study aims to identify the key metabolic compounds of supragingival plaque and potential metabolic pathways associated with severe early childhood caries (S-ECC) in preschool children, and to provide a basis for screening candidate biomarkers that could be beneficial to clinical practice guidelines. A cohort of 93 children, including 32 healthy controls (HC group), 31 children with S-ECC (SECC group) and 30 children with S-ECC and black stain (SECCBS group), were recruited. Supragingival plaques were collected for UHPLC-MS analyses followed by bioinformatics analysis. Differential metabolites (DMs) were screened through Partial Least Squares Discriminatory Analysis (PLS-DA) and enriched in KEGG pathways. Ecological network analysis and WGCNA were performed to construct co-expression networks. Ten machine learning (ML) models were optimized by feature selection and hyperparameter tuning, and evaluated using the area under the ROC curve (AUC), sensitivity, specificity, precision, and predictability comparison. A total of 1,069 metabolites were detected, with 497 annotated into 40 KEGG pathways, primarily amino acid and lipid metabolism. We identified 134 DMs between the HC and SECC groups, and 49 DMs between the SECC and SECCBS groups. WGCNA identified 32 co-expression modules in each paired comparison. In the HC-SECC comparison, the top correlated module pathways (neuroactive ligand-receptor interaction and pyrimidine metabolism) aligned precisely with the KEGG enrichment results of the DMs. In the SECC-SECCBS comparison, biosynthesis of unsaturated fatty acids and fatty acid degradation were uniquely enriched. For clinical prediction, the random forest (RF) model achieved the highest AUC (98.38) in the HC-SECC group, while the SVM-Poly model was superior in the SECC-SECCBS group (AUC = 95.10). The supragingival plaque metabolome is discernibly different among healthy children, those with severe caries, and those with black stain. Key metabolites including uracil, uridine 5’-diphosphate, cytosine, epinephrine, uridine, and deoxycytidylic acid are closely associated with dental caries occurrence and represent promising targets for early diagnosis. Additionally, Glycerophospho-N-palmitoyl ethanolamine (GP-NPEA) and lipid metabolic adaptations (such as unsaturated fatty acid pathways) in the SECCBS group provide a novel mechanistic explanation for the lower caries susceptibility and the pigment formation associated with black stain. These biomarkers offer valuable insights for precision risk stratification and personalized therapeutic strategies in pediatric dentistry.

## 1 Introduction

Dental caries, the most common oral disease among children, not only inhibits functions such as chewing and speech but also adversely affects maxillofacial development and general health (Baker et al., 2024). According to the ecological dysbiosis theory, dental caries is a result of the ecological dysfunction across the polymicrobial community through microbial interactions, resulting in local acidification and dental hard tissue demineralization followed by permanent damage to the tooth (Blostein et al., 2022; Shao et al., 2023). However, the composition and function of microbial plaque in children are highly dynamic, which challenge conventional detection methods.

Multi-omics technologies have provided a comprehensive insight into caries pathogenesis. High-throughput sequencing approaches such as 16S rRNA gene sequencing (Tak et al., 2025) and metagenomics (Gupta et al., 2024) enable comprehensive profiling of microbial diversity and genomic functional analysis in the evolution of dental caries, while mass spectrometry-based methods help uncover underlying molecular mechanisms by identifying metabolic shifts and associated pathways (Bauermeister et al., 2022; Ahmad et al., 2024). Pioneering studies have linked oral metabolite profiles to microbial activity: Takahashi et al. (2010) demonstrated glucose-induced changes in glycolytic intermediates in dental plaque, while Musalem-Dominguez et al. identified several salivary metabolites, such as glycine, taurine and propionate, as potential biomarkers in children with caries (Musalem-Dominguez et al., 2023). Integrated analyses further connected lactate and pyruvate (Kolderman et al., 2015) increase with *Streptococcus* and *Actinomyces* abundance (Moussa et al., 2022; Kim et al., 2023; Su et al., 2023), confirming their roles in dental caries. Notably, both lactate and pyruvate were widely acknowledged as metabolic markers for active dental caries, detecting in saliva and dental plaque (Kolderman et al., 2015; He et al., 2016; Li et al., 2022).

Interestingly, certain oral conditions such as black stain (BS) have been associated with altered metabolic activity and potentially reduced caries occurrence. Metagenomic analyses have revealed enriched carbohydrate and amino acid metabolism in BS-associated *Pseudopropionibacterium*, possibly contributing to plaque alkalinization and caries inhibition (Chen et al., 2021). Consistent with this, pathways including glycolysis/gluconeogenesis, cysteine and methionine metabolism, and pyruvate metabolism were more abundant in BS groups (Zhang et al., 2022). Nevertheless, the metabolic landscape across health, severe early childhood caries (S-ECC), and S-ECC with black stain remains unexplored.

Given the high-dimensional nature of metabolomic data, conventional statistics may overlook subtle but clinically relevant patterns. Machine learning (ML) provides a powerful alternative for identifying discriminative molecular signatures and predicting oral health status (Samaranayake et al., 2025). However, systematic comparisons of ML models using supragingival plaque metabolome data from children remain limited. Additionally, weighted gene co-expression network analysis (WGCNA) offers a robust framework for constructing co-expression networks and identifying key metabolite modules associated with clinical manifestations (Zhou et al., 2022). Although originally designed for transcriptomics, WGCNA’s mathematical algorithm seamlessly applies to high-dimensional metabolomics, where highly correlated metabolite modules can mathematically reflect tightly regulated enzymatic cascades and synchronized biochemical flux across complex polymicrobial communities. Yet, its application in pediatric caries metabolomics is still emerging.

In this study, we aim to delve into the oral metabolic perturbations across the health control (HC), SECC, and SECCBS groups, through an integrative analytical workflow including Partial Least Squares-Discriminant Analysis (PLS-DA), KEGG pathway enrichment, ecological network analysis, WGCNA, and machine learning, to uncover metabolite-based biomarkers and elucidate the molecular mechanisms for caries development.

## 2 Materials and methods

### 2.1 Patient recruitment

A total of 93 children aged 3 to 6 years were recruited in this study. Written parental consent was obtained from all guardians prior to participation. The study protocol was reviewed and approved by the Shenzhen Children’s Hospital Ethics Committee (No. 202319202). Exclusion criteria were as follows: (1) presence of systemic diseases; (2) visible enamel or dentin hypoplasia; (3) use of antibiotics or anti-inflammatory drugs within 2 weeks; and (4) receipt of fluoride treatment within the last month.

All participants were examined at the department of Stomatology, Shenzhen Children’s Hospital. Caries status was assessed by an experienced dentist using the decayed, missing, or filled teeth (dmft) index according to the World Health Organization (WHO) diagnostic criteria.

Participants were classified into three groups: healthy control (HC, n = 32), children without pigment who had no caries lesions or restorations; SECC (n = 31), children diagnosed with severe early childhood caries (S-ECC); SECCBS (n = 30), children presenting with extrinsic BS and meeting S-ECC criteria. The SECC and SECCBS groups included children with a decayed, missing, or dmft scores of ≥4 at age 3, ≥5 at age 4, or ≥6 at age 5. Non-cavitated lesions or white spot lesions were excluded from the HC groups.

### 2.2 Sampling

Supra-plaque samples were collected by a chief dentist between 9:00 and 10:00 a.m. Participants were instructed to abstain from morning toothbrushing until sample collection. Using a sterile dental excavator and collection containers, samples were pooled from the smooth labial surfaces of teeth. Immediately after collection, aliquots were prepared in 1.5 mL centrifuge tubes (Axygen, MCT-150-L-C-22, United States) and frozen at −80 °C within 2 h. All samples stored at −80 °C until further processing.

### 2.3 Untargeted metabolomics analysis of plaque samples

Ultra-High Performance Liquid Chromatography-Tandem Mass Spectrometry analyses were performed using an Orbitrap Q Exactive™ HF-X mass spectrometer (Thermo Fisher, Germany) in Novogene Co., Ltd. (Beijing, China). To minimize systematic bias during instrumental analysis, samples were analyzed in a randomized order. Quality control (QC) samples were interspersed at regular intervals throughout the sequence (one QC per five experimental samples). Samples were injected into a Hypersil Goldcolumn (100 mm × 2.1 mm, 1.9 μm) using a 12-min linear gradient at a flow rate of 0.2 mL/min. The eluents for the positive and negative polarity modes were eluent A (0.1% FA in Water) and eluent B (Methanol). The solvent gradient was set as follows: 2% B, 1.5 min; 2%–85% B, 3 min; 85%–100% B, 10 min; 100%–2% B, 10.1 min; 2% B, 12 min. Q Exactive™ HF-X mass spectrometer was operated to acquire data with spray voltage of 3.5 kV, capillary temperature of 320 °C, sheath gas flow rate of 35 psi and aux gas flow rate of 10 L/min, S-lens RF level of 60, Aux gas heater temperature of 350 °C.

### 2.4 Raw data processing and quality control and identification of differential metabolites

Raw mass spectrometry data were initially preprocessed to ensure quality, including peak picking, peak alignment, compound identification, and normalization using the MetaboAnalyst package v6.0 in R environment. Following data integrity verification, multivariate statistical analysis was conducted using Partial Least Squares-Discriminant Analysis (PLS-DA) to visualize group separation and access variable importance in projection (VIP). To balance statistical rigor and biological discovery, the differential metabolites (DMs) between the paired groups of HC vs. SECC, or SECC vs. SECCBS were identified by a multi-criteria screening strategy widely accepted in metabolomics studies (Tan et al., 2023; Lin et al., 2025), based on the following conditions: 1) the VIP score of more than 1; 2) fold-change (FC) of more than 1.2 or less than 0.833 as the threshold for biological relevance; and 3) *p*-value of less than 0.05 as the threshold for statistical significance. Volcano plots, generated with the ggplot2 package in R environment, were used to visualize and filter metabolites of interest based on log2 (fold change), -log10 (*p*-value), and VIP scores.

### 2.5 KEGG pathway enrichment

To identify biological pathways significantly associated with the DMs, Kyoto Encyclopedia of Genes and Genomes (KEGG, http://www.genome.jp/kegg) over-representation analysis (ORA) was performed using the clusterProfiler R package v4.14.6. The pre-filtered list of differential metabolites served as the input set, while all metabolites detected in the current untargeted metabolomics profiling that possessed assigned KEGG IDs were utilized as the background set. A hypergeometric test was applied to calculate the probability of observing at least the number of DMs mapped to that pathway by chance alone. Pathways with a *p*-value < 0.05 and *q* value < 0.05 were considered significantly enriched. The results were visualized using bar plots generated by the enrichplot R package, which illustrated the *p* value and metabolite counts for top 15 significantly enriched terms. This approach facilitated the prioritization of biologically relevant pathways across comparative groups, and only pathways with *p* values under a threshold of 0.05 were considered significant differences.

### 2.6 Gene set enrichment analysis (GSEA) enrichment analyses

Gene set enrichment analysis (GSEA) was performed to identify key biological pathways and core functional gene sets associated with the observed metabolic profiles, using the clusterProfiler R package v4.14.6. Enrichment analyses were conducted to determine whether a seria of priori-defined biological processes was enriched, with 1,000 label permutations and adjustment of false discovery rate (FDR) for significance testing. Pathway significance was determined using the normalized enrichment score (NES), which accounts for differences in gene set size and correlation with the expression profile. Gene sets with nominal *p*-value < 0.05 and *q* value < 0.25 were considered statistically significant. To enhance the robustness of findings, results from multiple comparative analyses were integrated, and only KEGG pathways consistently identified across different comparisons were retained as common signature pathways and core metabolites sets were analyzed.

### 2.7 Network analysis and key metabolites

Metabolite co-occurrence networks (CoNet) of were constructed using the ggClusterNet and phyloseq packages in R environment, with edges inferred via Spearman’s correlation analysis. An edge was established between two nodes (metabolites) only if it simultaneously satisfied two criteria: (1) absolute correlation coefficient |*r*| > 0.6, indicating a strong positive (co-occurrence) or negative (mutual exclusion) relationship; (2) *p*-value <0.05, ensuring the statistical significance of the correlation. Network visualization and topological analysis were conducted using Gephi software v0.9.233. Key topological properties were quantified, including number of nodes and edges, modularity, network diameter, average degree, centralization coefficient, clustering coefficient, connectance, and average path length. By ecological network analysis, networks were divided into different modules with nodes colored by module membership and sized proportionally to their connectivity degree. To identify key hub metabolites, *Zi-Pi* plot analysis was performed, which classified nodes with *Pi* > 0.62 and *Zi* > 2.5 as key network hubs.

### 2.8 Weighted gene co-expression network analysis (WGCNA)

The weighted metabolite co-expression network was constructed using the WGCNA R package v1.76. The soft-thresholding power was determined via the pickSoftThreshold function to ensure a scale-free topology. Metabolite modules were identified through hierarchical clustering based on a topological overlap matrix (TOM), followed by dynamic branch cutting using the cutreeDynamic function from the dynamicTreeCut package v1.63-1. Module eigengenes were calculated to represent each module’s expression profile, and eigengene adjacency was assessed to evaluate inter-module relationships. Based on co-expression relationships, the modules were distinguished well among one another. To identify clinically relevant modules, correlations between module eigengenes and clinical traits were computed,and modules with significant associations (*r* > 0.2, *p* < 0.05) were selected as an initial exploratory screening for further investigation. Within these modules, metabolites exhibiting module membership (MM) of more than 0.8 and metabolite significance (MS) of more than 0.2 were screened as candidate hub metabolites. Finally, the WGCNA network data were obtained and prepared for the subsequent analysis.

### 2.9 Machine learning predictions

Ten widely accepted machine learning models with least absolute shrinkage and selection operator logistic regression (LASSO), extreme gradient boosting (XGBoost), random forest (RF), K nearest neighbor (KNN), support vector machine (SVM), naïve Bayes classification (NBC), decision trees (Dtree) and multilayer perceptron (MLP) were employed to predict the final oral status in two paired groups, using R package of tidymodels v 1.30. Lasso serves as a fundamental linear model with interpretability, NBC is a probabilistic classifier with strong independence assumptions among features by applying Bayes’ theorem, and KNN is a simple instance-based learning algorithm preferring small dataset with meaningful local patterns. SVM and MLP are effective in high-dimensional predictive task by capturing complex patterns, while RF, XGBoost, and Dtree act as ensemble learning models, which enhance prediction accuracy by combining multiple learners to reduce variance and bias. Input data were organized as a feature matrix where rows represented individual samples and columns denoted metabolic features, with the final column serving as the target vector for binary clinical classification. To improve model interpretability and mitigating overfitting, a rigorous multi-step feature selection strategy was implemented to filter noise and irrelevant metabolites according the following excluding criteria: 1) low abundance (relative abundance < 0.0002); 2) low prevalence (detection rate < 10% across samples); 3) low variance (coefficient of variation [CV] < 0.1); 4) weak correlation with outcome (absolute correlation coefficient <0.2); 5) high multicollinearity (absolute correlation coefficient between features > 0.8). After feature selection, the relative abundances of metabolites were normalized and scaled to the range of 0–1. Using a stratified random sampling technique to preserve class balance within the two paired groups, 80% of the data were used as training dataset, and 20% were used as testing dataset at each stage of the resampling. Subsequently, the tidy model framework was leveraged to automate the hyperparameter tuning procedure, using the grid search strategy of five hundred random parameter sets. For each model, a comprehensive grid of potential hyperparameters was defined. The best hyperparameter combination was automatically selected based on resampling statistics, and the final model was fitted with these optimized parameters. To overfitting and ensure the robustness of the models, 10 repeated five-fold cross-validation was employed with the averaging performance metrics such as area under roc curve (AUC), sensitivity, specificity, and precision value calculated to determine the performance and generalization ability of the models.

### 2.10 Statistical analysis

Statistical analysis was performed using SPSS 26.0. Significant differences between two groups were analyzed by unpaired *t*-test and one-way ANOVA combined with a Student-Newman-Keuls (SNK) *post hoc* test. A *p* value of less than 0.05 was considered statistically significant.

## 3 Results

### 3.1 Metabolic profile summaries and annotation of KEGG pathway

**FIGURE 1:**
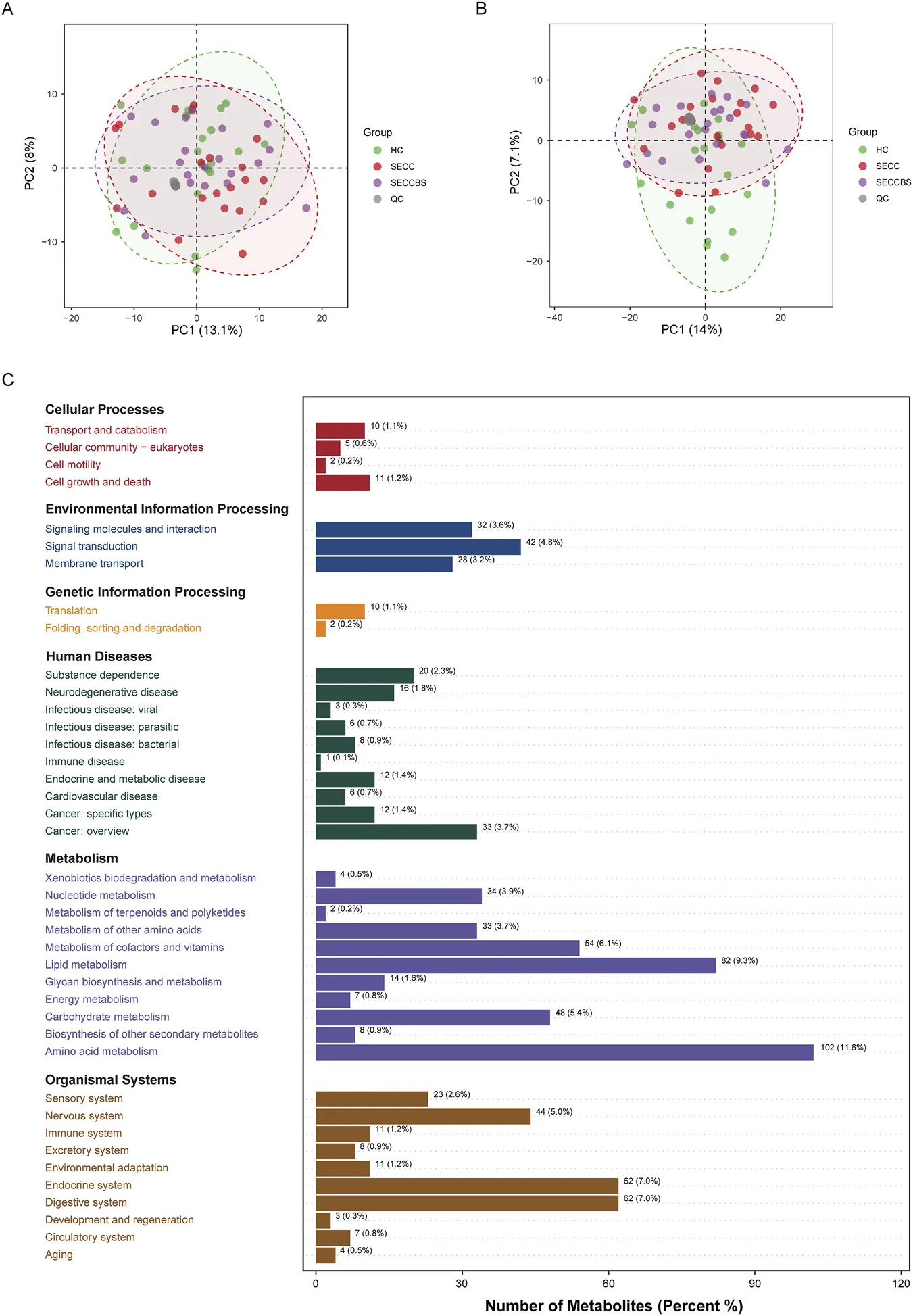
**(A,B)** Principal component analysis (PCA) of compounds captured with negative or positive polarity mode. The horizontal axis is the first principal component, and the vertical axis is the second principal component. The number in brackets is the score of the principal component, indicating the percentage of total variance explained by the corresponding principal component; **(C)** Bar chart of metabolite classification. X-axis represents the number of metabolites in each sub class, Y-axis represents the metabolite classification entries.

Principal component analysis (PCA) confirmed the stability and reproducibility of the analyses, as evidenced by the tight clustering and distinct separation of quality control (QC) replicates across QC, HC, SECC, and SECCBS samples (Figures 1A,B). As illustrated in Figure 1C, a total of 1,069 metabolites were identified, among which 497 were annotated and mapped to 40 KEGG pathways. The top four pathways, ranked in descending order based on the number of associated metabolites, were amino acid metabolism (11.6%), lipid metabolism (9.3%), digestive system (7.0%), and endocrine system (7.0%).

### 3.2 Differential metabolites in the paired groups

**FIGURE 2:**
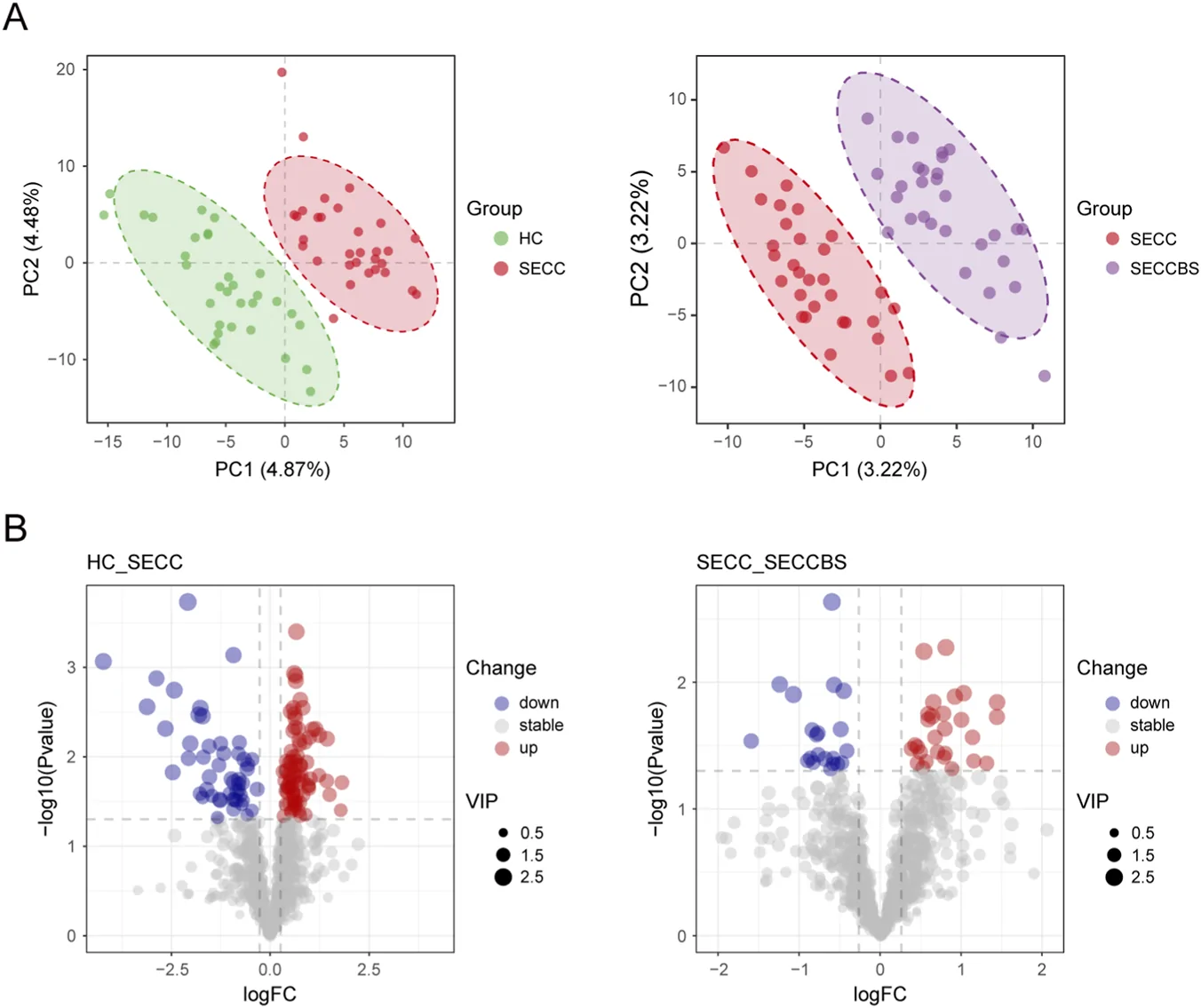
**(A)** PLS-DA results in the HC-SECC group and the SECC-SECCBS group. The horizontal axis is the first principal component, and the vertical axis is the second principal component. The number in brackets is the score of the principal component, indicating the percentage of total variance explained by the corresponding principal component. **(B)** Volcano plot analysis of metabolites between the paired groups (HC-SECC and SECC-SECCBS). Based on statistical significance (FC > 1.2 or <0.833, *p* value < 0.05 and VIP > 1), blue dot represents significantly down-regulated metabolites, and red dot represents significantly up-regulated metabolites, gray dot represents metabolites with no significant difference.

PLS-DA model exhibited evident clustering of samples by group (Figure 2A), indicating distinct metabolic profiles between the two paired groups. Moreover, the model quality was further validated by a 999-permutation test, which confirmed its robustness and predictive reliability. Comparison between the SECC and HC groups revealed 134 DMs, of which 84 were upregulated and 50 were downregulated (Figure 2B). As summarized in Supplementary Table S1, among the top five upregulated DMs, only one metabolite named phosphocholine was confirmed with biological roles, which was classified as organic nitrogen compounds and annotated to metabolic pathways (pathway ID, 01100, sub-class: Global and overview maps, class: Metabolism). Among the top 5 downregulated differential metabolites, melatonin, the only metabolite with recognized biological roles, was classified as Organic compounds and annotated to tryptophan metabolism (pathway ID, 00380, sub-class: Amino acid metabolism, class: Metabolism) and general metabolic pathways, respectively. Meanwhile, when the SECCBS group in comparison with the SECC group (Figure 2B), a total of 49 DMs were confirmed finally, including 28 upregulated and 21 downregulated metabolites. As listed in Supplementary Table S1, among the top five upregulated DMs, expert form melatonin, pilocarpine, classified as alkaloids and derivatives, belonged to the phytochemical compounds but could not be mapped to specific pathways. Similarly, among the top five downregulated DMs, 3-nitro-l-tyrosine, an organic acid derivative, also lacked pathway annotations. These findings underscored distinct DM patterns across the compared groups.

### 3.3 KEGG and GSEA pathway in enrichment analysis

**FIGURE 3:**
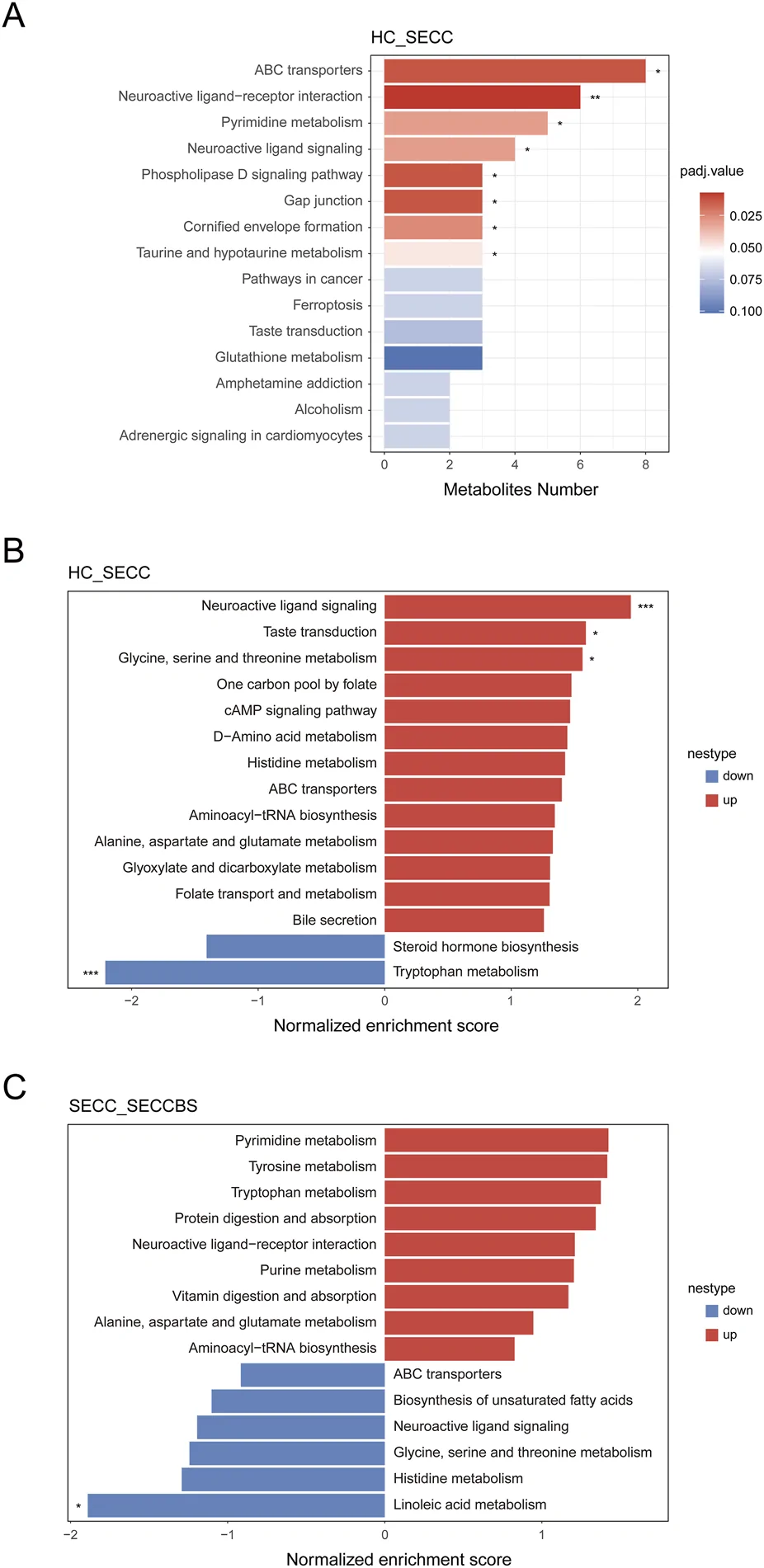
**(A)** Pathway enrichment of differential metabolites based on KEGG database. The horizontal axis represents the differential metabolite count in enriched pathway, and the vertical axis is the pathway enriched in HC-SECC group. **(B,C)** Pathway enrichment of metabolites in each paired group by GSEA analysis.

To characterize the functional classification of metabolites, a differential pathway analysis was performed using level 1 annotations of KEGG database. In the HC-SECC comparison, 4 KEGG pathways containing 50 differential metabolites were annotated. The four pathways ranked in descending order by metabolite count, were as follows: Environmental Information Processing (21 metabolites), Metabolism (8 metabolites), Organismal Systems (3 metabolites), and Cellular Processes (3 metabolites) (Figure 3A; Supplementary Table S2). These findings suggested that the metabolites with identification information detected and identified functioned mainly through two types of pathways, metabolism and organismal systems. However, no significant differences in the pathways were observed in the SECC-SECCBS group.

In contrast to KEGG pathway analysis, which typically relies on pre-filtering metabolites using specified significance thresholds (e.g., *p* < 0.05 and fold-change cutoffs) and thereby excluding compounds just below these limits, GSEA utilizes the entire metabolite dataset without applying a hard filter. This approach improves the detection of subtle but coordinated biological trends that might be overlooked under strict significance criteria. Therefore, we applied GSEA to further elucidate the underlying molecular mechanisms and identify key pathways along with their core metabolites. In the HC–SECC comparison (Figure 3B; Supplementary Table S3), four pathways were significantly enriched (*p* < 0.05): tryptophan metabolism; glycine, serine and threonine metabolism; neuroactive ligand signaling; and taste transduction. These results suggest that signaling molecules and interactions represent the most markedly perturbed processes during the occurrence and progression of dental caries. However, only the lipid metabolism pathway was enriched in the SECC-SECCBS comparison (Figure 3C; Supplementary Table S3), providing new insights into the molecular mechanisms driving oral disease development.

### 3.4 Co-occurrence networks and keystone

**FIGURE 4:**
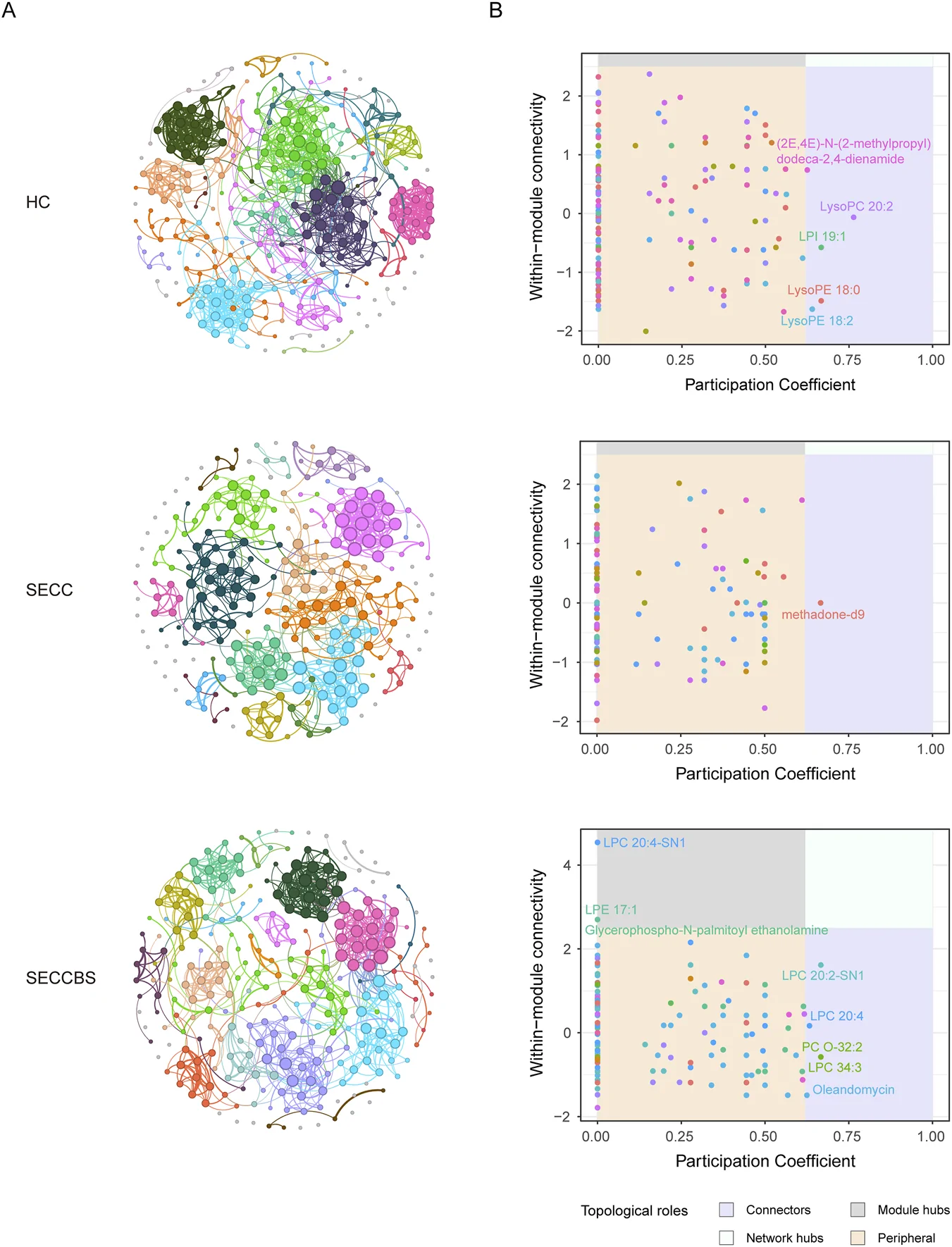
**(A)** Molecular ecological network (MEN) analysis; **(B)** *Zi-Pi* method to scale key hubs of dental plaque at different depth. *Zi*, the within-module connectivity, describes the connectivity of a node with other nodes in the same module, and the participation coefficient, *Pi*, reflects the degree of connectivity of a node with different modules. Except for Peripheral compounds (*Zi* ≤ 2.5, *Pi* ≤ 0.62), other metabolites are considered as key metabolites: (1) Connectors metabolites (*Zi* ≤ 2.5, *Pi* > 0.62); (2) Module metabolites (*Zi* > 2.5, *Pi* ≤ 0.62); (3) Network metabolites (*Zi* > 2.5, *Pi* > 0.62).

Based on molecular ecological network (MEN) analysis, topological indices were calculated to characterize the complexity and potential metabolic interactions across microbial communities at different ecological depths. From an ecological standpoint of the oral metabolome (Figure 4A; Table 1), the SECCBS network exhibited the most key hubs of 13, compared to 10 in the HC network and 6 in the SECC network. Notably, the clustering coefficient increased significantly in the SECC group to 0.419, representing 1.08- and 1.12-fold increases over the HC and SECCBS groups, respectively. With respect to network modularity, clusters decreased in both the SECC and SECCBS groups relative to the HC group, with a more compressive reduction in the SECC group, implying diminished reciprocal interactions among metabolome. Centralization betweenness, an indicator of interaction strength, was lowest in the SECC network, further supporting weakened network connectivity. In addition, the HC network displayed a high proportion of positive correlations among nodes (94.24%), indicating that metabolites achieved a strong cooperation. In contrast, the SECCBS network showed an elevated negative correlation proportion (8.38%), reflecting a shift from a synergistic to a competitive metabolic interaction.

**TABLE 1:** Topological features of the oral metabolome co-occurence networks.

Topological features	HC	BSCF	SECCBS	SECC
Edges^a^	608	507	556	461
Positives	573	496	513	427
Negatives	35	11	43	34
Vertices	288	260	272	255
Connectance (edge_density)^b^	0.0147	0.0151	0.0151	0.0142
average.degree (Average K)^c^	4.222	3.9	4.088	3.616
average.path.length^d^	3.277	3.19	3.152	2.888
Diameter^e^	8.97	7.983	9.131	7.327
Clustering coefficient (Average.CC)^f^	0.387	0.373	0.373	0.419
no.clusters^g^	61	50	61	68
centralization.degree^h^	0.055	0.039	0.088	0.0566
centralization.betweenness^i^	0.081	0.074	0.142	0.076
RM (relative.modularity)^j^	0.691	0.551	0.475	0.28
The number of keystone nodes^k^	10	8	13	6

^a^ Edges represent the number of connections/correlations.

^b^ Connectance(edge_density) is to quantify how densely interconnected a network is.

^c^ Average degree is the average number of links per node.

^d^ Average path length represents the average number of steps (edges) required to travel along the shortest path between any two nodes in the network.

^e^ Network diameter is the shortest path between the two most separated nodes.

^f^ Clustering coefficient is the degree to which nodes in a network tend to form clusters.

^g^ The number of cluster is a measure of the network’s fragmentation or modularity.

^h^ Centralization degree measures the extent to which the network’s connectivity is dominated by a few highly connected nodes, as opposed to being evenly distributed across all nodes.

^i^ Betweenness centrality measures the extent to which a node lies on the shortest paths between other nodes, acting as a bridge or intermediary.

^j^ Modularity is the strength of division of a network into modules.

^k^ Nodes represents bacterial taxa with co-ocurrence correlation Spearman >0.5 or <−0.5.

In addition, ecological network analysis was performed to unveil metabolites interactions and reveal the key hubs in the network. As depicted in Figure 4B, five key hubs were (2E,4E)-N-(2-methylpropyl) dodeca-2,4-dienamide, LysoPC 20:2, LPI 19:1, LysoPE 18:0 and LysoPE 18:2 (*Pi* > 0.62) in the HC network, as eight key hubs in the SECCBS network that included LPC 20:2-SN1, LPC 20:4, PC O-32:2, Oleandomycin, and LPC 34:3 (*Pi* > 0.62), LPC 20:4-SN1, LPE 17:1 and Glycerophospho-N-palmitoyl ethanolamine (GP-NPEA) (*Zi* > 2.5). Yet, only one compound named Methadone-d9 existed in the SECC network (*Pi* > 0.62), potentially indicative of its cumulative effect through the ecological modulation of keystone metabolites.

**FIGURE 5:**
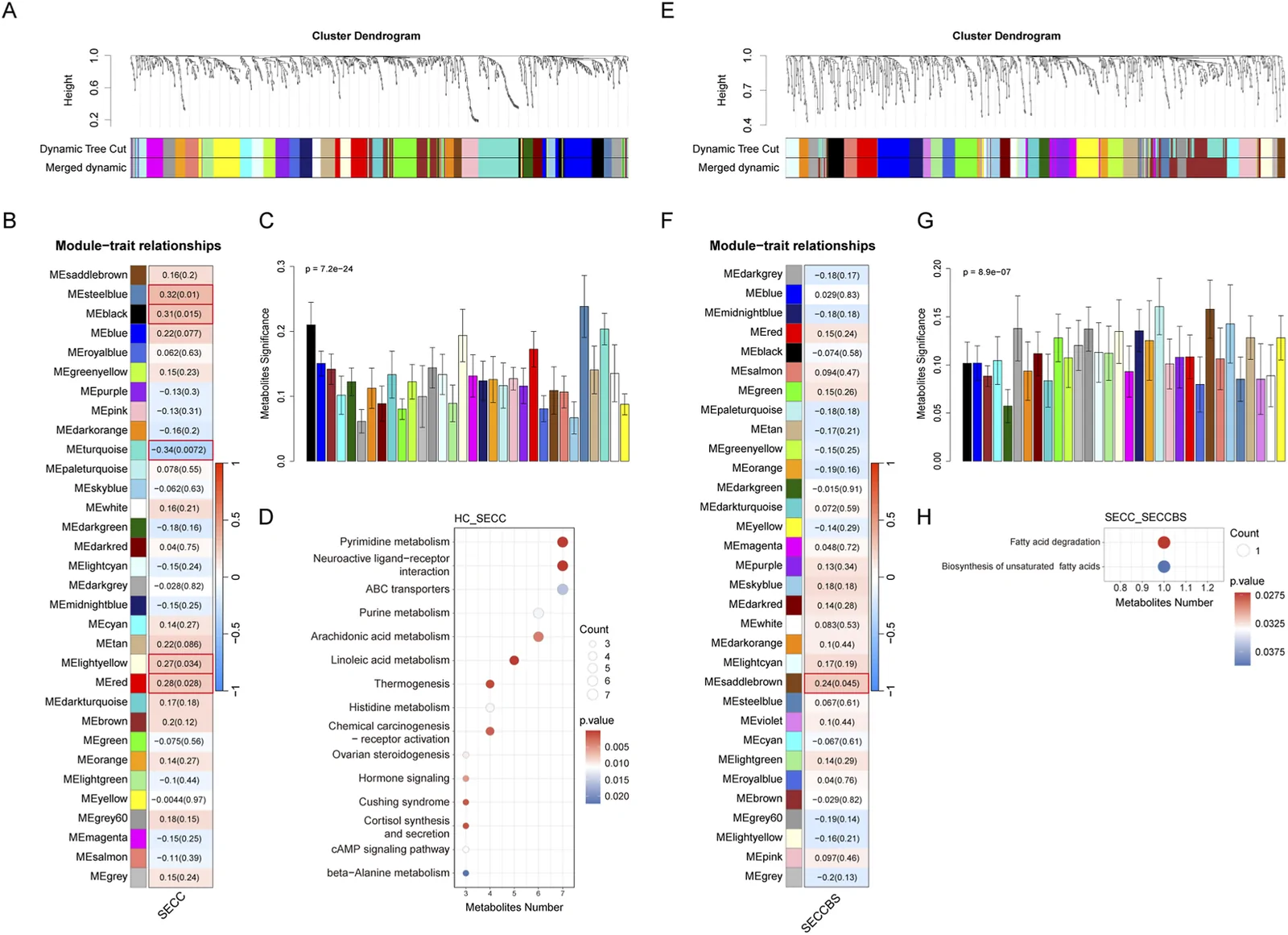
WGCNA network analysis. **(A,E)** Cluster dendrogram of metabolites with dissimilarity based on topological overlap in the HC-SECC group **(A)** and the SECC-SECCBS group **(E)**. The different color row below the dendrogram represents module membership clustered by the dynamic tree cut method. **(B,F)** Module-trait relationship analysis between the paired HC-SECC groups **(B)** and the paired SECC-SECCBS groups **(F)**. The colors of columns represent the correlation coefficients for module-trait relationship. Red represents positive correlation, whereas blue represents negative correlation. Each column was marked with the exact number of correlation coefficient and *p* value in bracket for module- trait relationship. **(C,G)** Bar chart of metabolite significance across the different modules in the paired HC-SECC groups **(C)** and the paired SECC-SECCBS groups **(G)**. **(D,H)** Pathway enrichment of modules significantly associated with specific trait in the paired HC-SECC groups **(D)** and the paired SECC- SECCBS groups **(H)**. The X-axis represents the metabolite number enriched in metabolic pathway. The dot size represents the count of metabolites annotated to this pathway. Enrichment analysis was based on metabolites in significant modules and KEGG database.

To further investigate functionally significant metabolites, WGCNA network with optimized soft-threshold auto-selected for fit a network of scale-free, was utilized to conduct the in-depth analysis on significance key-hub metabolites. Hierarchical clustering based on co-expression relationships identified 32 modules in both the paired HC-SECC and SECC-SECCBS groups, each color-coded (Figures 5A,B,E,F). The gray module was reserved for metabolites with low intramodular connectivity that could not be assigned to other modules. As delineated in Figure 5B, module-trait relationship analysis identified five co-expression modules with a prominent association with SECC in the HC-SECC comparison, which comprising four positive and one negative correlation. In contrast, only one module exhibited a positive correlation with the SECCBS phenotype in the SECC–SECCBS comparison. The trait-associated modules in both comparative groups exhibited significantly higher MS values compared to other modules (Figures 5C,G).

To gain functional insights into these relationships, we performed KEGG pathway enrichment analysis on the metabolites within the significant modules. In the HC–SECC comparison, metabolites from the correlated modules were enriched in 13 pathways. The top four pathways, ranked by metabolite count, were neuroactive ligand–receptor interaction, pyrimidine metabolism, arachidonic acid metabolism, and linoleic acid metabolism (Figure 5D; Supplementary Table S4). Notably, a striking concordance was observed, with the top two pathways aligning precisely with the enrichment results of KEGG on the DMs, strongly implicating these pathways as pivotal players in the pathophysiology of dental caries.

Interestingly, in the SECC–SECCBS comparison, metabolites from the significant module were enriched in only two pathways: biosynthesis of unsaturated fatty acids and fatty acid degradation (Figure 5H; Supplementary Table S4). This distinct profile implied a specific link between black stain formation and perturbations in fatty acid metabolism, which provided a novel perspective on the mechanism foundation for the anti-caries properties of black stain.

**FIGURE 6:**
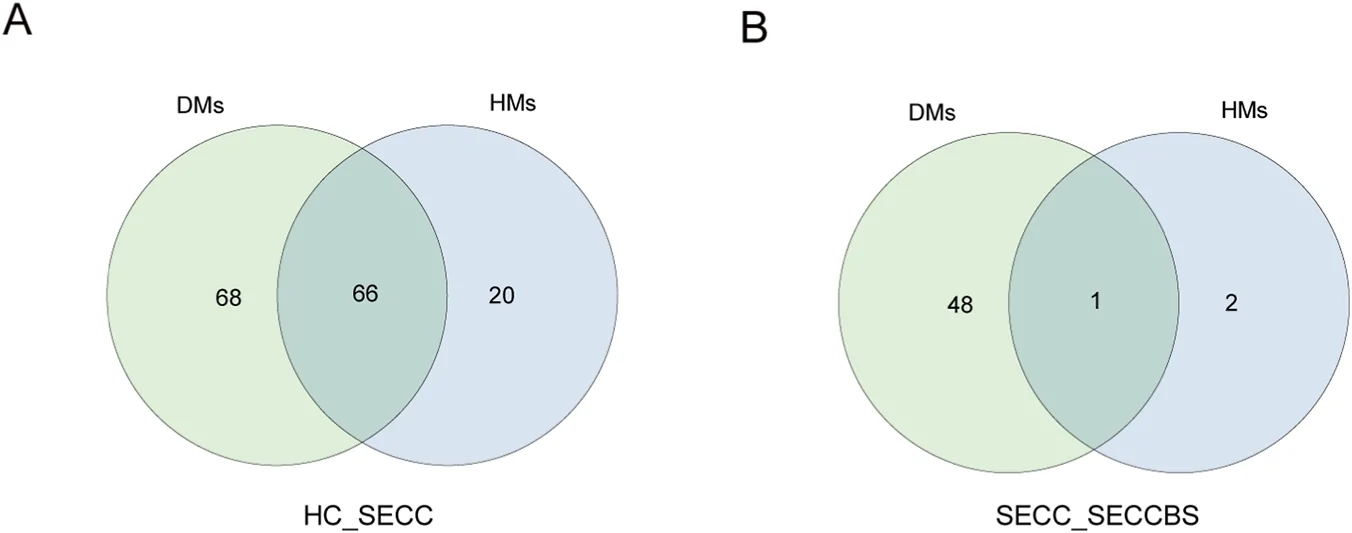
Venn diagram of differential metabolites (DMs) and hub metabolites between HC vs SECC **(A)** and SECC vs SECCBS **(B)**.

To assess the concordance between DMs and key hub metabolites identified via WGCNA across the comparison groups, we integrated the respective datasets for subsequent analysis (Supplementary Table S5). In the HC–SECC comparison (Figure 6A; Supplementary Table S6), 63 overlapped metabolites were observed between DMs and key hub metabolites. Additionally, 71 metabolites were unique to the DMs, while only 15 compounds were exclusively identified as key hub metabolites. For the SECC–SECCBS comparison (Figure 6B; Supplementary Table S6), only one metabolite was common to both DMs and key hub metabolites, with 48 metabolites specific to the DMs and 2 metabolites uniquely assigned as key hub metabolites.

### 3.5 Machine learning prediction

**FIGURE 7:**
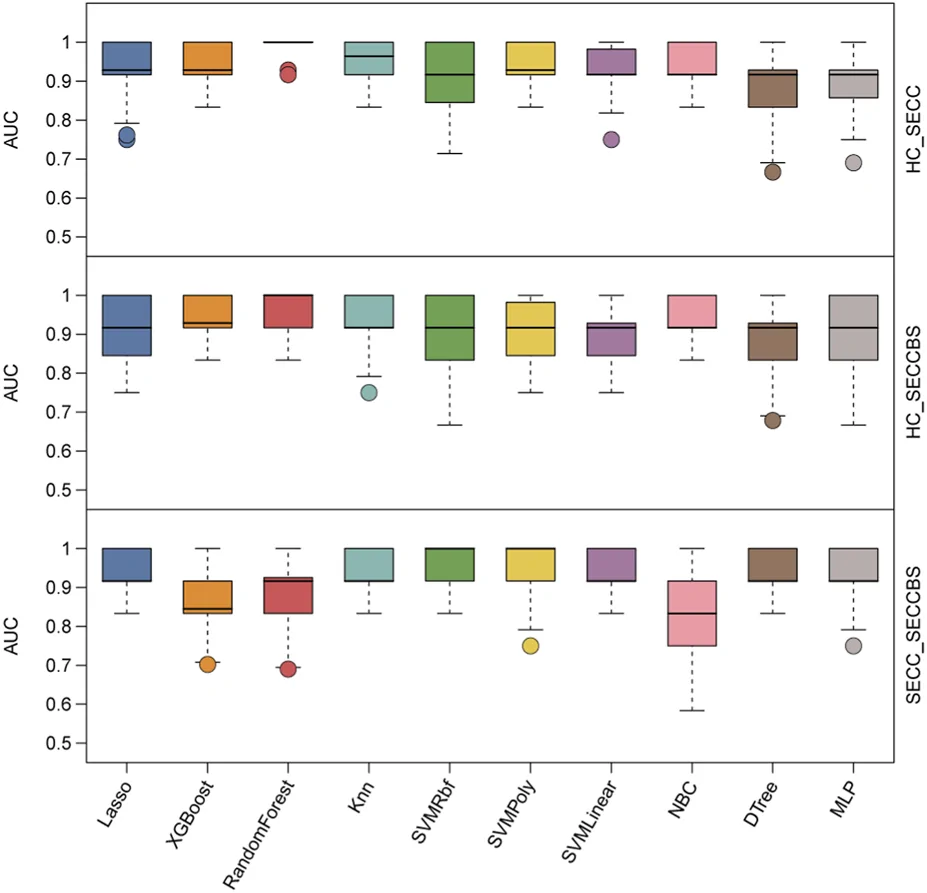
Distinctive prediction of classification using ten machine learning models.

To enable intelligent clinical stratification and prediction among distinct patient subgroups, ten machine learning models were constructed through the molecular features and metabolic shifts. These models were systematically evaluated and compared in the field of area under the curve (AUC) as performance metrics. As summarized in Table 2 and illustrated in Figure 7, model performance varied across comparison groups. The random forest (RF) model was more suitable for binary classification prediction of the HC-SECC group, achieving the highest AUC of 98.38. Conversely, the SVM-Poly model attained the highest prediction performance for the SECC-SECCBS group (AUC = 95.10). These findings suggest that differences in model performance may reflect underlying variations in data structure and metabolic characteristics across patient groups, which might offer a novel insight for the diagnosis and therapeutic strategies for tooth decay. Conversely, while the AUC is widely accepted as a robust aggregate metric, other metrics such as sensitivity, specificity, precision, and etc., remain pivotal for model selection and evaluation, particularly within specific clinical diagnostic contexts where distinct performance characteristics are required.

**TABLE 2:** Comparison of the models for the prediction of favorable outcomes.

Models	HC-SECC				HC-SECCBS				SECC-SECCBS			
	AUC	Sensitivity	Specificity	Precision	AUC	Sensitivity	Specificity	Precision	AUC	Sensitivity	Specificity	Precision
Lasso	93.55±6.76 [91.63, 95.47]	93.62±9.84 [90.82, 96.42]	93.48±8.09 [91.18, 95.78]	94.16±7.36 [92.07, 96.26]	91.81±7.60 [89.65, 93.97]	93.62±8.43 [91.22, 96.02]	90.00±12.60 [86.42, 93.58]	91.83±9.84 [89.03, 94.63]	95.00±4.76 [93.65, 96.35]	100.00±0.00 [100.00, 100.00]	90.00±9.52 [87.29, 92.71]	91.79±7.69 [89.60, 93.97]
XGBoost	93.71±5.93 [92.03, 95.40]	93.81±7.70 [91.62, 96.00]	93.62±7.93 [91.37, 95.87]	94.26±7.17 [92.23, 96.30]	95.07±5.29 [93.57, 96.57]	96.81±6.46 [94.97, 98.65]	93.33±9.52 [90.63, 96.04]	94.57±7.58 [92.41, 96.72]	86.90±7.92 [84.65, 89.16]	87.14±12.60 [83.56, 90.72]	86.67±13.04 [82.96, 90.37]	88.49±10.44 [85.52, 91.45]
RF	98.38±3.28 [97.45, 99.31]	100.00±0.00 [100, 100]	96.76±6.56 [94.90, 98.63]	97.32±5.43 [95.78, 98.86]	96.67±5.05 [95.23, 98.10]	100.00±0.00 [100.00, 100.00]	93.33±10.10 [90.46, 96.20]	94.79±7.66 [92.61, 96.97]	88.64±8.34 [86.27, 91.01]	87.29±11.86 [83.92, 90.66]	90.00±12.14 [86.55, 93.45]	91.16±10.45 [88.19, 94.13]
KNN	95.26±5.38 [93.73, 96.79]	93.76±9.55 [91.05, 96.48]	96.76±6.56 [94.90, 98.63]	97.15±5.79 [95.51, 98.80]	93.33±6.94 91.36, 95.31	100.00±0.00 [100.00, 100.00]	86.67±13.88 [82.72, 90.61]	90.60±9.86 [87.26, 92.86]	93.33±6.07 [91.61,95.06]	100.00±0.00 [100.00, 100.00]	86.67±12.14 [83.22, 90.12]	89.48±9.22 [86.86, 92.11]
SVMlinear	92.024±6.08 [90.30, 93.75]	87.29±11.63 [83.98, 90.59]	96.76±6.56 [94.90, 98.63]	97.07±5.95 [95.38, 98.76]	90.33±6.73 [88.42, 92.25]	90.67±9.97 [87.83, 93.50]	90.00±10.10 [87.13, 92.87]	91.37±8.48 [88.96, 93.78]	95.02±5.04 [93.59, 96.46]	96.71±6.65 [94.83, 98.60]	93.33±9.52 [90.63, 96.04]	94.62±7.52 [92.48, 96.75]
SVMpoly	93.74±5.65 [92.13, 95.34]	90.71±9.94 [87.89, 93.54]	96.76±6.56 [94.90, 98.63]	97.13±5.81 [95.48, 98.78]	90.33±7.87 [88.10, 92.57]	90.67±9.39 [88.00, 93.33]	90.00±11.66 [86.69, 93.31]	91.57±9.65 [88.83, 94.32]	95.10±6.26 [93.32, 96.87]	96.86±6.37 [95.05, 98.67]	93.33±11.17 [90.16, 96.51]	94.56±8.92 [92.02, 97.10]
SVMrbf	90.67±8.46 [88.26, 93.07]	84.57±15.31 [80.22, 88.92]	96.76±6.56 [94.90, 98.63]	96.79±6.53 [94.93, 98.65]	88.81±9.20 [86.19, 91.42]	87.62±13.54 [83.77, 91.47]	90.00±10.65 [86.97, 93.03]	90.75±9.47 [88.06, 93.44]	95.07±6.00 [93.37, 96.78]	96.81±6.46 [94.97, 98.65]	93.33±8.91 [90.80, 95.87]	94.15±7.71 [91.96, 96.35]
NBC	95.101±5.13 [93.64, 96.55]	100.00±0.00 [100.00, 100.00]	90.19±10.26 [87.28, 93.11]	92.18±8.07 [89.89, 94.48]	93.48±6.37 [91.67, 95.29]	96.95±6.18 [95.20, 98.71]	90.00±10.65 [86.97, 93.03]	91.79±8.37 [89.41, 94.17]	81.74±10.45 [78.77, 84.71]	93.48±10.53 [90.48, 96.47]	70.00±17.17 [65.12, 74.88]	77.44±10.68 [74.40, 80.48]
DTree	88.81±8.36 [86.43, 91.19]	93.62±8.61 91.17, 96.07]	84.00±14.57 [79.86, 88.14]	87.14±10.96 [84.02, 90.25]	88.67±8.36 [86.29, 91.04]	90.67±10.53 [87.68, 93.66]	86.67±12.14 [83.22, 90.12]	88.66±9.31 [86.01, 91.31]	93.36±5.32 [91.85, 94.87]	96.71±6.65 [94.83, 98.60]	90.00±10.10 [87.13, 92.87]	91.79±8.13 [89.48, 94.10]
MLP	90.62±6.46 [88.78, 92.46]	87.62±11.28 [84.41, 90.83]	93.62±8.43 [91.22, 96.02]	93.92±8.31 [91.56, 96.28]	90.40±8.31 [88.04, 92.77]	87.48±12.52 [83.92, 91.04]	93.33±10.10 [90.46, 96.20]	94.10±8.69 [91.63, 96.57]	93.38±6.29 [91.59, 95.17]	93.43±8.81 [90.92, 95.93]	93.33±8.25 [90.99, 95.68]	94.02±7.51 [91.88, 96.15]

Lasso, selection operator (LASSO) logistic regression; XGBoost, Extreme gradient boosting; RF, random forest; KNN, K nearest neighbor; SVM, support vector machine; NBC, Naïve Bayes classification; DTree, Decision trees; MLP, machine learning potential.

## 4 Discussion

Dental caries is a multifactorial, ecological disease. To date, the oral metabolome has emerged as a direct readout of oral microbiota function, offering critical insights into the outcomes of dysbiosis, but its underlying mechanisms remain not fully elucidated. Moreover, individuals reared under similar conditions exhibit varying susceptibility to caries, suggesting that differences in oral metabolite profiles may contribute to this variation in susceptibility. In this study, we conducted the first metabolomic analysis comparing profile shifts across the HC, SECC, and SECCBS groups.

In our data, citrulline and glutamate were significantly more abundant in the cariogenic environment, aligning with earlier metabolomic reports (Wu et al., 2022; Li et al., 2023). These studies demonstrated that arginine deiminase system constitutes a key pathway utilized by various bacteria, including *Streptococci,* to counteract acid-induced demineralization. In this system, arginine is deiminated to citrulline, yielding ammonia, ornithine, carbon oxides, and ATP.

Pathway enrichment analysis of differential metabolites identified 7 KEGG Level 2 metabolic pathways that were altered between the HC and SECC groups. ABC transporters, which mediate the transport of carbohydrates, amino acids, proteins, lipids, and inorganic ions, play an essential role in biofilm formation (Benda et al., 2021). Given that dental plaque biofilm development and maturation are critical to caries initiation, shifts in the oral microbiome that influence ABC transporter activity may promote cariogenesis. Indeed, both S-ECC and recurrent caries have been linked to ABC transporters (Kalpana et al., 2020). ABC transporters are multidomain membrane proteins capable of exporting a wide variety of substrates, utilizing energy derived from ATP binding and hydrolysis (Beis and Rebuffat, 2019). Therefore, it is of great importance to further elucidate the role of ABC transporters in biofilm formation and their role as a prerequisite for the development of caries. Uracil, uridine 5′-diphosphate (C00015), cytosine, uridine (C00299), and deoxycytidylic acid were enriched in pyrimidine metabolism, which was in accordance with a study reported by Pereira et al. (2019). This suggests that oxidative stress and inflammation may accelerate purine degradation. A similar mechanism may occur in dental caries, potentially as part of a localized inflammatory response, leading to elevate hypoxanthine and uracil levels. Coincidentally, the pyrimidine metabolism and ABC transporters were also certified by KEGG pathway enrichment analysis of the metabolites from the significant modules through this study.

In the present study, Glutamine and glutamate (Glu-Gln) was identified as one of the most significant compounds. Consistent with other studies (Washio et al., 2016; Liu et al., 2023), who stated that the patients with caries presented a shift change of Glu-Gln in the SECC group. It is the main source of ammonia production at high concentrations, contributes to pH homeostasis and acid neutralization, prevents acid-induced enamel demineralization. Simulatively, DMs showed that upregulated levels of epinephrine were detected in the active caries group and annotated to the signaling molecules and interaction, which was consistent with prior reports (Kolderman et al., 2015; Kondo et al., 2019). Epinephrine regulates salivary amylase secretion, which helps reduce plaque acids produced by *Streptococcus mutans*. Since these acids contribute to enamel dissolution, salivary amylase may serve as a biomarker for caries risk (Culp et al., 2021). Indeed, α-amylase activity is closely associated with caries experience, and low levels of α-amylase may increase susceptibility to ECC (Parsaie et al., 2022).

In this study, the application of WGCNA successfully bridged individual marker screening with system-level pathway architecture. The remarkable concordance between the top module pathways derived from WGCNA and individual DMs enrichment strongly reinforces the validity of this network-based approach. It proves that WGCNA does not introduce statistical artifacts but rather enhances the biological interpretability of the plaque metabolome by identifying functional co-response clusters that shift in unison during the health-to-caries transition. To identify common metabolites, an integrated analysis was performed between DMs and key hub metabolites derived from WGCNA. In the significant black module of HC-SECC group (*p* = 0.015), palmitoleic acid (C08362) was enriched. It is universally acknowledged that cell membrane fatty acids play a crucial role in maintaining normal cellular physiological functions. When cells are subjected to environmental stresses such as temperature, ions, salts, drugs, and oxidative stress, they can rapidly alter the fatty acid composition of their cell membranes, changing their morphology or enhancing their migration ability to resist damage from the external environment (Upchurch, 2008). In cariogenic bacteria, membrane fatty acids maintain the acid-base balance within and outside the cell membrane. At pH 5, the proportion of long-chain monounsaturated fatty acids in the cell membrane of *S. mutans* increases due to acid stress (Quivey et al., 2000). Under acidic conditions, the content of long-chain monounsaturated fatty acids in the cell membrane of *S. mutans* increases. Furthermore, after induction of fluoride-resistant *S. mutans*, the increase in monounsaturated fatty acid content is greater than that in the parent strain with enhanced acid resistance (Zhu et al., 2024). Vice versa, when compared to the SECC-SECCBS group in the saddlebrown module (*p* = 0.045), metabolites from the significant module were enriched in only two pathways: biosynthesis of unsaturated fatty acids and fatty acid degradation. This distinct metabolic signature suggests a specific association between black stain formation and disruptions in fatty acid metabolism, offering a previously unrecognized perspective on the mechanistic basis for the cariostatic properties of black stain. From a physiological standpoint, the concurrent enrichment of these two pathways reflects a sophisticated metabolic adaptation and cell membrane remodeling process within the supragingival plaque of the SECCBS group. Under the acidic pressure of a severe cariogenic environment, oral microorganisms rapidly adjust their membrane lipid composition to sustain cell viability. The upregulation of unsaturated fatty acid biosynthesis enhances the proportion of long-chain monounsaturated fatty acids in the lipid bilayer, hereby neutralizing intracellular acidification. Concurrently, the activation of fatty acid degradation pathways implies that black stain-associated bacteria actively utilize hydrophobic lipid components as alternative bioenergetic substrates. This metabolic shift could reduce the microbial reliance on carbohydrate fermentation, subsequently diminishing localized lactic acid production and contributing to the lower caries susceptibility observed in children with black stain. Furthermore, altered lipid fluxes might cross-talk with glycerophospholipid networks to modulate local melanogenesis pathways, facilitating the phenotypic presentation of extrinsic dark pigments. This intricate balance between lipid synthesis and catabolism underscores a resilient, non-cariogenic ecological niche within the dysbiotic polymicrobial community.

BS has been widely attributed to the formation of sulfide compounds, particularly ferric sulfide (Dong et al., 2024). Consistent with the findings of Zhang et al. (2022). The current study also detected elevated levels of methionine in the BS group. Both sulfur-containing amino acids serve as potential substrates for hydrogen sulfide (H_2_S) production via microbial fermentation (Stipanuk, 2020). This aligns with the present results and further suggests that methionine was modulated by the digestive system and may concurrently influence the progression of dental caries and tooth black stain. Involved the progression of dental caries and tooth black stain simultaneously. Moreover, the SECCBS co-expression network exhibited a greater number of key hubs, reflecting higher network complexity. GP-NPEA, is widely acknowledged as a key lipid mediator associated with cellular stress responses and apoptosis regulation (Deng et al., 2025). It is the metabolic precursor of palmitoyl ethanolamide (PEA) (Duan et al., 2022), classified to the categories of oxygen compounds, was mainly enriched in glycerophospholipid metabolism, arachidonic acid metabolism, linoleic acid metabolism, alpha-linoleic acid metabolism, and melanogenesis in metabolome, suggesting that these pathways may play important roles in the formation of melanogenesis (Hu et al., 2025), which is consistent with observations in this study. Furthermore, the elevated negative correlation ratio among nodes in the SECCBS network implies a shift from cooperative to competitive metabolic interactions. This altered dynamic may partly explain why children with dental black stain exhibit lower susceptibility to dental caries (Mousa et al., 2022).

We have carefully rephrased statements to ensure that metabolic differences are framed as “associated shifts” or “consequences/markers of dysbiosis” rather than direct causes of dental caries or black stain. The molecular segregation between SECC and SECCBS established in this study provides critical translational frameworks that extend far beyond theoretical microbiology into chairside pediatric dental practice. Primarily, our findings pave the way for precision risk stratification. Historically, extrinsic black stain was managed purely as a cosmetic concern, despite clinical observations linking it to lower caries susceptibility. By mapping the unique metabolic architecture of SECCBS, which was characterized by the upregulation of acid-buffering unsaturated fatty acid synthesis and specific glycerophospholipid cascades, clinicians can move toward biomarker-driven screening. Pediatric patients can be stratified into distinct cariogenic risk categories at an earlier stage, allowing for personalized preventive protocols; S-ECC patients with highly acidogenic profiles require aggressive, high-frequency fluoride interventions, whereas SECCBS patients may benefit from dynamic monitoring focused on maintaining their inherent “ecological buffer”.

AI technology has potential applications in pedodontics for detection and prediction toolkits, including the detection and classification of supernumerary teeth, dental anomalies, early childhood caries, pit and fissure sealants, dental caries, dental plaque, and ectopic eruptions (Alharbi and Alharbi, 2024). Park et al. (2021) confirmed that both traditional logistic regression and ML-based models such as XGBoost, RF and Light GBM algorithms were powerful classifiers in S-ECC. Ramos-Gomez et al. (2021) proposed using a machine learning technique called RF to predict the presence of active caries and generate the optimal set of questions, helping predict dental caries. In addition, analysis of ROC, AUC, and accuracy demonstrated that the logistic regression model combined with salivary cystatin concentrations and birth weight exhibited the strongest and clinically acceptable capacity to distinguish between children with S-ECC and HC controls (Koopaie et al., 2021). In the current study, ten ML models were optimized by feature selection and hyperparameter tuning, and evaluated with averaging performance metrics such as AUC, sensitivity, specificity, and precision value. In comparison of the different ML models, RF obtained the highest accuracy in the HC-SECC group, as SVMPoly was superior in the SECC-SECCBS group. The discrepancy between this outcomes and earlier study may be attributed to variations in population characteristics, datasets, and disease features. Further research is warranted to develop a more practical S-ECC prediction model for clinical use.

## 5 Conclusion

The metabolome associated with carious lesions or tooth black stain was discernibly different. These outcomes provide novel mechanistic insights into the mechanism and offer promising candidate biomarkers for the precision medicine in the management of tooth decay. Future larger investigations will be required to verify and corroborate our findings in larger cohorts.

### 5.1 Limitations

Several limitations should be acknowledged in the present study. Firstly, given the exploratory nature and limited sample size, both differential metabolite screening and WGCNA module-trait correlation analyses were performed using nominal *p*-values (*p* < 0.05). This approach prioritizes minimizing Type II errors to preserve potential relevant biological insights. Although the risk of false positives was rigorously mitigated through a multi-criteria screening strategy and downstream orthogonal cross-validation, the reported associations should be interpreted with caution and require validation in larger, independent cohorts. Secondly, although the machine learning models demonstrated promising predictive performance, the lack of an external validation cohort limits the generalizability of our findings. Future multi-center studies encompassing larger, diverse populations are warranted to confirm the external validity and clinical utility of these models. Moreover, given the exploratory and cross-sectional nature of this study, the observed metabolic differences represent correlations rather than causal relationships. It remains ambiguous whether these metabolic alterations are the upstream drivers of cariogenesis or the downstream consequences of the carious lesions and microbial dysbiosis. Our analysis did not adjust for potentially significant confounding variables, including individual dietary habits (e.g., sugar consumption frequency), specific oral hygiene practices, and socioeconomic status, which could independently modulate the supragingival plaque metabolome. Finally, given our distinct findings regarding the enrichment of unsaturated fatty acid biosynthesis and degradation pathways in the SECCBS group, further mechanistic research should be directed toward the decoupling of microbial lipid metabolism and clinical presentation. Specifically, *in vitro* biofilm models and meta-transcriptomic coupling are required to elucidate how specific keystones interact with ferric sulfide pathways to competitively suppress cariogenic proliferation or alter biofilm cell membrane permeability under acid stress.

## Data Availability

The data reported in this paper have been deposited in the OMIX, China National Center for Bioinformation / Beijing Institute of Genomics, Chinese Academy of Sciences (https://ngdc.cncb.ac.cn/omix accession no.OMIX018082; Project: PRJCA067562).
